# Proportional Assist Ventilation Improves Leg Muscle Reoxygenation After Exercise in Heart Failure With Reduced Ejection Fraction

**DOI:** 10.3389/fphys.2021.685274

**Published:** 2021-06-21

**Authors:** Audrey Borghi-Silva, Cassia da Luz Goulart, Cláudia R. Carrascosa, Cristino Carneiro Oliveira, Danilo C. Berton, Dirceu Rodrigues de Almeida, Luiz Eduardo Nery, Ross Arena, J. Alberto Neder

**Affiliations:** ^1^Cardiopulmonary Physiotherapy Laboratory, Federal University of São Carlos (UFSCar), São Paulo, Brazil; ^2^Pulmonary Function and Clinical Exercise Physiology Unit, Division of Respiratory Diseases, Department of Medicine, Federal University of Sao Paulo (UNIFESP), São Paulo, Brazil; ^3^Post-Graduation Program on Collective Health, Federal University of Juiz de Fora, Juiz de Fora, Brazil; ^4^Pulmonary Physiology Unit, Federal University of Rio Grande do Sul, Porto Alegre, Brazil; ^5^Division of Cardiology, Department of Medicine, Federal University of Sao Paulo (UNIFESP), São Paulo, Brazil; ^6^Department of Physical Therapy, College of Applied Health Sciences, University of Illinois at Chicago, Chicago, IL, United States; ^7^Respiratory Investigation Unit, Division of Respirology, Department of Medicine, Kingston Health Science Center and Queen’s University, Kingston, ON, Canada

**Keywords:** blood flow, heart failure, non-invasive ventilation, hemodynamics, exercise recovery

## Abstract

**Background:**

Respiratory muscle unloading through proportional assist ventilation (PAV) may enhance leg oxygen delivery, thereby speeding off-exercise oxygen uptake ($\dot{\text{V}}\,{\text{O}}_{2}$) kinetics in patients with heart failure with reduced left ventricular ejection fraction (HFrEF).

**Methods:**

Ten male patients (HFrEF = 26 ± 9%, age 50 ± 13 years, and body mass index 25 ± 3 kg m^2^) underwent two constant work rate tests at 80% peak of maximal cardiopulmonary exercise test to tolerance under PAV and sham ventilation. Post-exercise kinetics of $\dot{\text{V}}\,{\text{O}}_{2}$, vastus lateralis deoxyhemoglobin ([deoxy-Hb + Mb]) by near-infrared spectroscopy, and cardiac output (Q_*T*_) by impedance cardiography were assessed.

**Results:**

PAV prolonged exercise tolerance compared with sham (587 ± 390 s vs. 444 ± 296 s, respectively; *p* = 0.01). PAV significantly accelerated $\dot{\text{V}}\,{\text{O}}_{2}$ recovery (*τ* = 56 ± 22 s vs. 77 ± 42 s; *p* < 0.05), being associated with a faster decline in Δ[deoxy-Hb + Mb] and Q_*T*_ compared with sham (*τ* = 31 ± 19 s vs. 42 ± 22 s and 39 ± 22 s vs. 78 ± 46 s, *p* < 0.05). Faster off-exercise decrease in Q_*T*_ with PAV was related to longer exercise duration (*r* = −0.76; *p* < 0.05).

**Conclusion:**

PAV accelerates the recovery of central hemodynamics and muscle oxygenation in HFrEF. These beneficial effects might prove useful to improve the tolerance to repeated exercise during cardiac rehabilitation.

## Introduction

The rate at which oxygen uptake ($\dot{\text{V}}{\text{O}}_{2}$) decreases after dynamic exercise has been used to assess disease severity and prognosis and, more recently, the effectiveness of interventions in patients with heart failure with reduced ejection fraction (HFrEF) (Guazzi et al., 2004; Dall’Ago et al., 2006; Compostella et al., 2014; Georgantas et al., 2014; Fortin et al., 2015; Bailey et al., 2018). Although oxygen (O_2_) delivery is usually in excess of the decreasing O_2_ demands during recovery from exercise in normal subjects, this might not be the case in HFrEF, a phenomenon that helps to explain why the evaluation of exercise recovery kinetics has gained popularity in the clinical arena (Kemps et al., 2009; Poole et al., 2012).

Exercise recovery kinetics have been shown to be more reproducible than those at the onset of exercise, and less influenced by oscillatory breathing or the confounding effects of a prolonged “cardiodynamic” phase I (Francis et al., 2002; Kemps et al., 2007). Moreover, activities of daily living are characterized by their short-term and repetitive nature, thereby suggesting that fast recovery from effort is important for the successful completion of any subsequent task (Hirai et al., 2019).

In this context, a previous study has shown that unloading the respiratory musculature with proportional assist ventilation (PAV) was associated with improved peripheral muscle oxygenation during constant-load exercise, as indicated by blunted changes in Δ deoxyhemoglobin ([deoxi-Hb + Mb]) determined by near-infrared spectroscopy (NIRS) and longer exercise tolerance in patients with HFrEF (Borghi-Silva et al., 2008a), chronic obstructive pulmonary disease (COPD) (Borghi-Silva et al., 2008b), and HFrEF-COPD coexistence (da Luz Goulart et al., 2020).

Interestingly, inspiratory muscle training associated with whole-body training also improved the cardiorespiratory responses to exercise, leading to a faster $\dot{\text{V}}{\text{O}}_{2}$ recovery in HFrEF (Dall’Ago et al., 2006). Based on the previous evidence indicating that post-exercise $\dot{\text{V}}{\text{O}}_{2}$ kinetics can be accelerated by interventions focused on improving O_2_ delivery (Borghi-Silva et al., 2008a), this study hypothesized that, compared to sham ventilation, the rate of increase in muscle reoxygenation would be accelerated by PAV in HFrEF. Confirmation of this hypothesis indicates that the beneficial effects of respiratory muscle unloading on leg O_2_ delivery are not limited to the onset of exercise (Borghi-Silva et al., 2008a), lending support to the notion that $\dot{\text{V}}{\text{O}}_{2}$ recovery kinetics are clinically useful to assess the efficacy of interventions in this patient population.

## Materials and Methods

### Subjects and Design

The current study cohort included 10 non-smoking male patients who were recruited from the HFrEF outpatient clinic of the Institution (Miocardiopathy Ambulatory, Division of Cardiology). Patients with HFrEF satisfied the following inclusion criteria: (1) diagnosis of HFrEF documented for at least 4 years; (2) three-dimensional echodopplercardiography showing left ventricular ejection fraction (LVEF) <35%; (3) New York association functional class II and III; and (4) no hospitalizations in the previous 6 months. All patients were optimally treated according to the American Heart Association/American College of Cardiology treatment recommendations for stage “C” patients (i.e., reduced LVEF and current or previous symptoms of heart failure) (Hunt et al., 2005). All patients were judged to be clinically stable and compensated on medical therapy at the time of evaluation. In addition, patients were familiarized with stationary bicycle cardiopulmonary exercise tests prior to data collection.

Patients were excluded from study if they (1) demonstrate evidence of obstructive pulmonary disease [forced expiratory volume in 1 s (FEV_1_)/forced vital capacity (FVC) ratio of <70%]; (2) have a history of smoking; (3) have a history of exercise-induced asthma; (4) have unstable angina or significant cardiac arrhythmias; (5) have anemia (hemoglobin <13 g%); (6) had myocardial infarction within the previous 12 months; (7) have primary valvular heart disease, neuromuscular or musculoskeletal disease, or other potential causes of dyspnea or fatigue; or (8) had participated in cardiovascular rehabilitation in the preceding year. Patients gave a written informed consent, and the study protocol was approved by the Institutional Medical Ethics Committee (CEP 0844/06).

### Study Protocol

Subjects performed a ramp-incremental cardiopulmonary exercise test (CPX) on a cycle ergometer (5–10 W/min) to determine $\dot{\text{V}}{\text{O}}_{2}$ at peak exercise. These loads were individually adjusted according to the severity of symptoms and the severity of the disease. On a separate day, subjects performed a high-intensity constant work rate (CWR) trial test at 80% peak workrate (WR) to individually select PAV’s flow and volume assist levels. At a subsequent experimental visit, the patients undertook, 1 h apart, two CWR at the previously defined WR to the limit of tolerance (*Tlim*, s*).* Data were also recorded during the 5-min of passive recovery (without any muscle contraction), which followed exercise. During these tests, patients were randomly assigned to receive sham ventilation and the pre-selected levels of PAV. The patients and the accompanying physician were unaware of the ventilation strategy (PAV or sham) under use. This was accomplished by visually isolating the ventilator and its monitor from both the physician’s and the patient’s view. Vastus lateralis muscle oxygenation levels were assessed by NIRS. In addition, systemic O_2_ delivery was followed by continuous monitoring of exercise cardiac output (transthoracic impedance) and metabolic and ventilatory measurements were collected breath-by-breath.

### Non-invasive Positive Pressure Ventilation

PAV was applied *via* a tight-fitting facial mask with pressure levels being delivered by a commercially available mechanical ventilator (Evita-4; Draeger Medical, Lübeck, Germany). PAV is a non-invasive modality that provides flow (FA, cmH_2_O L^–1^ s^–1^) and volume assistance (VA, cmH_2_O/L) with the intent of unloading the resistive and elastic components of the work of breathing. PAV levels were individually set on a preliminary visit using the “run-away” method: the protocols for adaptation at rest and exercise were as previously described (Younes, 1992; Bianchi et al., 1998; Carrascossa et al., 2010). Sham ventilation was applied *via* the same equipment using the minimal inspiratory pressure support of 5 cmH_2_O; moreover, 2 cmH_2_O of positive end-expiratory pressure was used to overcome the resistance of the breathing circuit (Borghi-Silva et al., 2008a,b). Both PAV and sham were delivered with an O_2_ inspired fraction of 0.21.

### Maximal and Submaximal Cardiopulmonary Exercise Testing

Symptom-limited CPX was performed on a cycle ergometer using a computer-based exercise system (CardiO_2_ System^TM^ Medical Graphics, St. Paul, MN). Breath-by-breath analysis ventilatory expired gas analysis was obtained throughout the test. Incremental adjustment of work rate was individually selected (usually 5–10 W/min). The load increment was individually selected based on the symptoms of dyspnea reported by the patient for some physical activities and the experience of the research team. In patients with more severe symptoms such as dyspnea to walk on level ground, the load increase was 5 W, while those who did not report fatigue for this activity, an increase of 10 W was selected, which is considered to test completion ideally between 8 and 12 min (Neder et al., 1999). The carbon dioxide (CO_2_) and O_2_ analyzers were calibrated before and immediately after each test using a calibration gas (CO_2_ 5%, O_2_ 12%, and N_2_ balance) and a reference gas [room air after ambient temperature and pressure saturated (ATPS) to standard temperature and pressure, dry (STPD) correction]. A Pitot tube (Prevent Pneumotach^TM^, MGC) was calibrated with a 3-L volume syringe by using different flow profiles. As a bi-directional pneumotachograph based on turbulent flow, the Pitot tube was adapted at the opening of the mask used for non-invasive ventilation.

The following data were recorded: $\dot{\text{V}}{\text{O}}_{2}$ (ml/min), $\dot{\text{V}}{\text{CO}}_{2}$ (ml/min), minute ventilation (${\dot{\text{V}}}_{E}$, L/min), and the partial pressure of end-tidal CO_2_ (P_*ET*_CO_2_) (mmHg). Ventilatory efficiency (${\dot{\text{V}}}_{E}$/$\dot{\text{V}}{\text{CO}}_{2}$ slope) was defined as the ventilatory response relative to CO_2_ production. The ${\dot{\text{V}}}_{E}$/$\dot{\text{V}}{\text{CO}}_{2}$ slope provides the ventilatory requirements to wash out metabolically produced CO_2_ (Keller-Ross et al., 2016). Peak $\dot{\text{V}}{\text{O}}_{2}$ was the highest 15-s averaged value at exercise cessation (Neder et al., 1999). In addition, 12-lead electrocardiographic monitoring was carried out throughout testing. Subjects were also asked to rate their “shortness of breath” at exercise cessation using the 0–10 Borg’s category-ratio scale, and symptom scores were expressed in absolute values and corrected for exercise duration. Capillary samples were collected from the ear lobe for blood lactate measurements (mEq/L) at rest and at exercise cessation (Yellow Springs 2.700 STAT plus^TM^, Yellow Springs Instruments, OH, United States).

### Skeletal Muscle Oxygenation

Skeletal muscle oxygenation profiles of the left *vastus lateralis* were evaluated using a commercially available NIRS system (Hamamatsu NIRO 200^TM^, Hamamatsu Photonics KK, Japan) during the CWR tests with PAV and sham (Borghi-Silva et al., 2008b). Previously, the skin under the probe was shaved in the dominant thigh. The skinfold was < 12.5 mm in all patients to ensure that the amount of fat between the muscle probe did not interfere with the signals (van der Zwaard et al., 2016). The light probe was placed to the belly of the vastus lateralis muscle, approximately 15 cm from the upper edge of the patella, and firmly attached to the skin using adhesive tape (Goulart et al., 2020b) and involved in a black closed mesh with a velcro. Briefly, one fiberoptic bundle carries the NIR light produced by the laser diodes to the tissue of interest while a second fiberoptic bundle returns the transmitted light from the tissue to a photon detector in the spectrometer. The intensity of incident and transmitted light is recorded continuously and, together with the relevant specific extinction coefficients, used for online estimation and display of the changes from the resting baseline of the concentrations of [deoxy-Hb + Mb] (Borghi-Silva et al., 2008b). [Deoxy-Hb + Mb] levels were obtained second-by-second at rest, during exercise, and 5 min of recovery. [Deoxy-Hb + Mb] has been used as a proxy of fractional O_2_ extraction in the microcirculation, reflecting the balance between O_2_ delivery and utilization (Sperandio et al., 2009). In order to reduce intrasubject variability and improve intersubject comparability, [deoxy-Hb + Mb] values were expressed as the percentage of the maximal value determined on a post-exercise maximal voluntary contraction (MVC) after 5-min recovery. This study used a single probe consisting of eight laser diodes operating at two wavelengths (690 and 830 nm). Due to the uncertainty of the differential pathlength factor (DPF) for the quadriceps, we did not use a DPF in the present study. The distance between the light emitters and the receiver was 3.5 cm (Goulart et al., 2020b).

### Central Hemodynamics

Cardiac output (Q_*T*_, L/min) was measured using a calibrated signal-morphology impedance cardiography device (PhysioFlow PF-05, Manatec Biomedical, France). The PhysioFlow principle is based on the assumption that variations in impedance occur when an alternating current of high frequency (75 kHz) and low magnitude (1.8 mA) passes through the thorax during cardiac ejection (Borghi-Silva et al., 2008a). In preliminary experiments, the system detected small changes in Q_*T*_ (∼0.1 L/min) with acceptable accuracy (within ± 10% for all readings) (Borghi-Silva et al., 2008a). The values were recorded as delta (Δ) from baseline and expressed relative (%) to the amplitude of variation from baseline to the steady-state with sham ventilation (within ± 2 standard deviations of the local mean).

### Kinetics Analysis

Breath-by-breath $\dot{\text{V}}{\text{O}}_{2}$, Δ[deoxy-Hb + Mb], HR, and Q_*T*_ data were time aligned to the cessation of exercise and the first 180 s of recovery were interpolated second by second (SigmaPlot 10.0 Systat Software Inc., San Jose, CA, United States). Data were analyzed from the last 30 s of exercise to obtain a more stable baseline and over the 180 s of recovery; i.e., it is considered only the primary component of the response. Using this approach, it was assured that the same amount of data was included in the kinetic analysis of $\dot{\text{V}}{\text{O}}_{2}$, Δ [deoxy-Hb + Mb], and Q_*T*_ for each intervention, minimizing model-dependent effects on results. The model used for fitting the kinetics response was:

(1) [Y](t)=[Y](ss)--A⋅(1-e)-(t-TDρ)/τ

where the subscripts “ss” and “ρ” refer to steady-state and primary component, respectively. “A,” “TD,” and “*τ*” are the amplitude, time delay, and time constant of the exponential response of the interest (i.e., ∼time to reach 63% of the response following the end of exercise), respectively. The overall kinetics of Δ[deoxy-Hb + Mb] were determined by the mean of response time (MRT = *τ* + TD) (Mazzuco et al., 2020).

### Statistical Analysis

The required number of patients to be assessed (*n* = 10, crossover study) was calculated considering the *τ* (s) of $\dot{\text{V}}{\text{O}}_{2}$ during PAV and sham in HF patients as the main outcome (Mazzuco et al., 2020), assuming a risk of α of 5% and β of 20%. The SPSS version 13.0 statistical software was used for data analysis (SPSS, Chicago, IL, United States). According to data distribution, results were reported as mean ± SD or median and ranges for symptom scores. The primary end point of the study was changes in MRT-[deoxi-Hb + Mb] with PAV compared to sham. Secondary end points included Tlim, changes of τ $\dot{\text{V}}{\text{O}}_{2}$, and Q_*T*_ recovery kinetics. To contrast differences between PAV and sham on exercise responses and kinetic measurements, non-paired *t* or Mann–Whitney tests were used as appropriate. Pearson’s product moment correlation was used to assess the level of association between continuous variables. The level of statistical significance was set at *p* < 0.05 for all tests.

## Results

All patients completed the maximal and submaximal exercise tests. Baseline characteristics of HFrEF patients are presented in Table 1. The LVEF ranged from 22 to 26%. Peak WR and $\dot{\text{V}}{\text{O}}_{2}$ of all patients were below the age- and gender-corrected lower limits of normality (Neder et al., 1999). Eight patients were Weber class C and two were class B. As anticipated by long-term β-blocker therapy, patients presented with a reduced peak HR response.

**TABLE 1:** Patient characteristics at rest, medication used, and cardiopulmonary exercise testing data (*N* = 10).

Anthropometric characteristics	
Age (years)	50 ± 13
Height (m)	1.67 ± 0.05
Body mass (kg)	74 ± 13
Body mass index (kg/m^2^)	25 ± 3
**Echocardiography**	
LVEF (%)	26 ± 4
**Etiology of heart failure**	
Ischemic	3
Non-ischemic	7
**Medications**	
Diuretic	9
Digitalis	3
Carvedilol	10
Angiotensin-converting enzyme inhibitor	8
**Maximal exercise**	
Power, W	90 ± 27
**Metabolic**	
Peak $\dot{\text{V}}{\text{O}}_{2}$,% pred	55 ± 14
Peak $\dot{\text{V}}{\text{O}}_{2}$, ml min	1.222 ± 284
Peak $\dot{\text{V}}{\text{O}}_{2}$, ml min^–1^ kg^–1^	17 ± 5
Peak $\dot{\text{V}}{\text{CO}}_{2}$, ml min	1.358 ± 436
Peak blood lactate (mmol/L)	4.0 ± 2.1
**Ventilatory**	
${\dot{\text{V}}}_{E}$, L min^–1^	47 ± 8.4
Respiratory rate, breaths min^–1^	32 ± 8
V_*T*_, L	1.60 ± 0.39
**Cardiovascular**	
Heart rate, bpm	116 ± 15
Heart rate,% pred	65 ± 5.8
Oxygen pulse (ml min^–1^/bpm)	11 ± 1.5
**Subjective**	
Dyspnea scores	6 ± 2.0
Leg effort scores	6 ± 2.1

LVEF, left ventricle ejection fraction of left ventricle; FEV_1_, forced expiratory volume in 1 s; $\dot{\text{V}}{\text{O}}_{2}$, oxygen consumption; $\dot{\text{V}}{\text{CO}}_{2}$, carbon dioxide output; ${\dot{\text{V}}}_{E}$, minute ventilation; V_*T*_, tidal volume, HR, heart rate. *Mean (SD).

### Physiological Responses at the Tlim After Sham vs. PAV

The values selected for volume and flow assist during PAV were 5.6 ± 1.4 cmH_2_O/L and 3.0 ± 1.2 cmH_2_O L^–1^ s^–1^, respectively. PAV significantly improved exercise tolerance as shown by a longer *Tlim* compared to sham ventilation (*p* < 0.05, Table 2). There was no significant change in $\dot{\text{V}}{\text{O}}_{2}$ at *Tlim*; however, a significantly higher $\dot{\text{V}}{\text{CO}}_{2}$ was observed with PAV (*p* < 0.05, Table 2). In addition, ventilatory efficiency improved with PAV as demonstrated by a significant reduction in ${\dot{\text{V}}}_{E}$/$\dot{\text{V}}{\text{CO}}_{2}$ slope compared to sham ventilation (*p* < 0.05, Table 2).

**TABLE 2:** Main physiological responses at the time of constant work rate exercise tolerance (Tlim) after sham or proportional assist ventilation (*N* = 10).

	Sham	PAV	*p*-value
Tlim (s)	444 ± 296	587 ± 390	0.01
$\dot{\text{V}}{\text{O}}_{2}$ (ml/min)	1,183 ± 450	1,280 ± 285	0.40
$\dot{\text{V}}{\text{CO}}_{2}$ (ml/min)	1,153 ± 287	1,258 ± 257	0.03
RER	1.02 ± 0.08	1.02 ± 0.36	0.96
${\dot{\text{V}}}_{E}$ (L/min)	44.6 ± 7.1	45.6 ± 6.4	0.67
${\dot{\text{V}}}_{E}$_/_CO_2_ slope	40.3 ± 10.7	37.1 ± 7.6	0.04
P_*ET*_CO_2_, mmHg	32.1 ± 7.5	33.3 ± 7.1	0.54
HR, bpm	109 ± 11	110 ± 15	0.70
HR,% peak	63 ± 6	64 ± 7	0.72
Oxygen pulse (ml/min/beat)	10.8 ± 4.4	11.7 ± 2.6	0.42
Dyspnea (0–10)	5.7 ± 1.4	5.1 ± 1.7	0.19
Leg effort (0–10)	5.9 ± 3.0	5.4 ± 2.5	0.49
Δlactate (peak-rest, mmol/L)	2.10 ± 1.16	1.88 ± 1.14	0.67

**$\dot{\text{V}}{\text{O}}_{2}$, oxygen consumption; $\dot{\text{V}}{\text{CO}}_{2}$, carbon dioxide output; RER, respiratory exchange ratio; ${\dot{\text{V}}}_{E}$, minute ventilation; P_*ET*_CO_2_, end-tidal partial pressure for CO_2_; HR, heart rate. p < 0.05 (paired t or Wilcoxon tests for between-group differences at a given time point). Values are means ± SD.**

### Off-Exercise Dynamics After Sham and PAV

**FIGURE 1:**
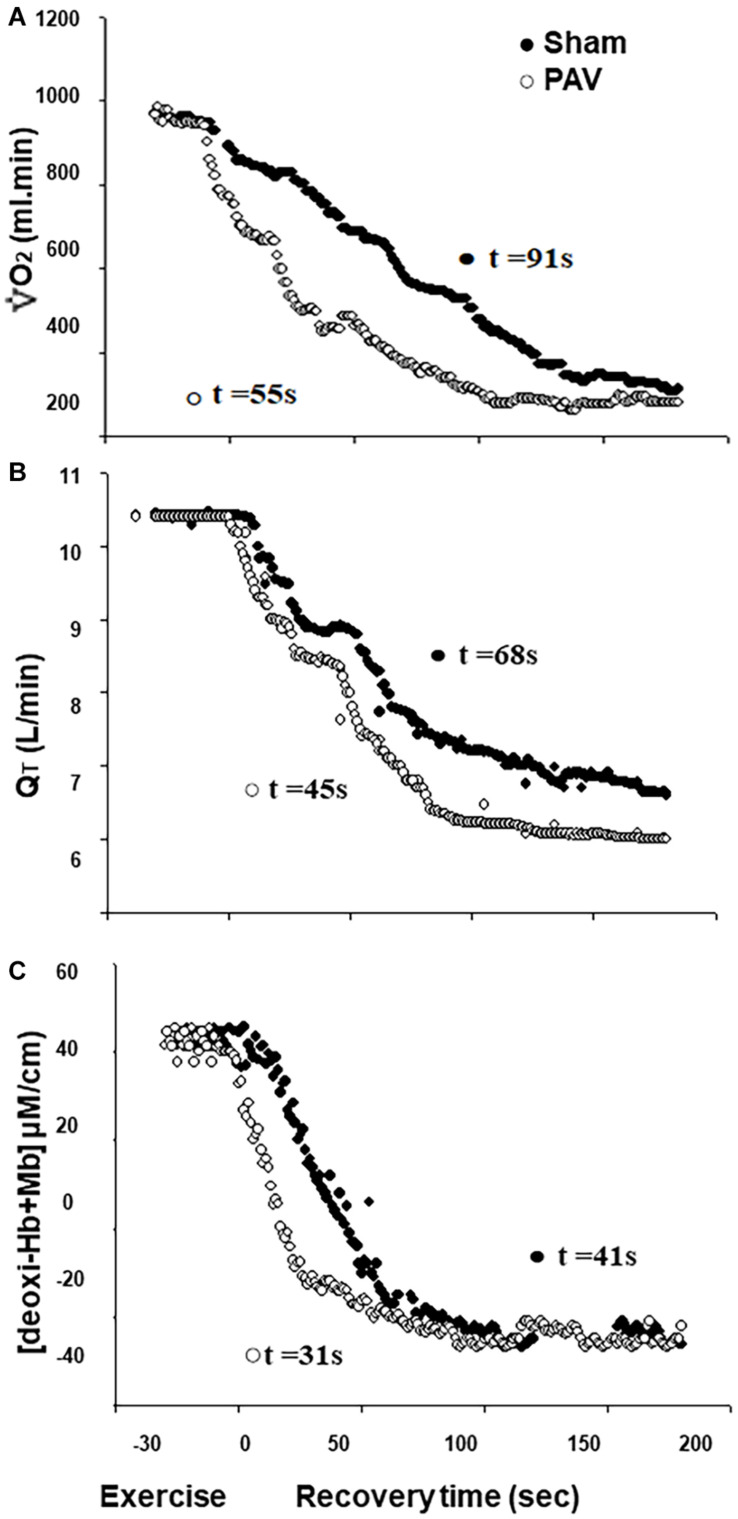
Pulmonary O_2_ uptake [${\dot{\text{V}}\text{O}}_{2p}$, **(A)**], cardiac output [Q_*T*_, **(B)**], and deoxy-hemoglobin concentration [deoxy-Hb + Mb, **(C)**] off-kinetics variables at high-intensity constant workload exercise test of a representative patient with HFrEF contrasting PAV (⭘) vs. Sham Ventilation (⬤).

**FIGURE 2:**
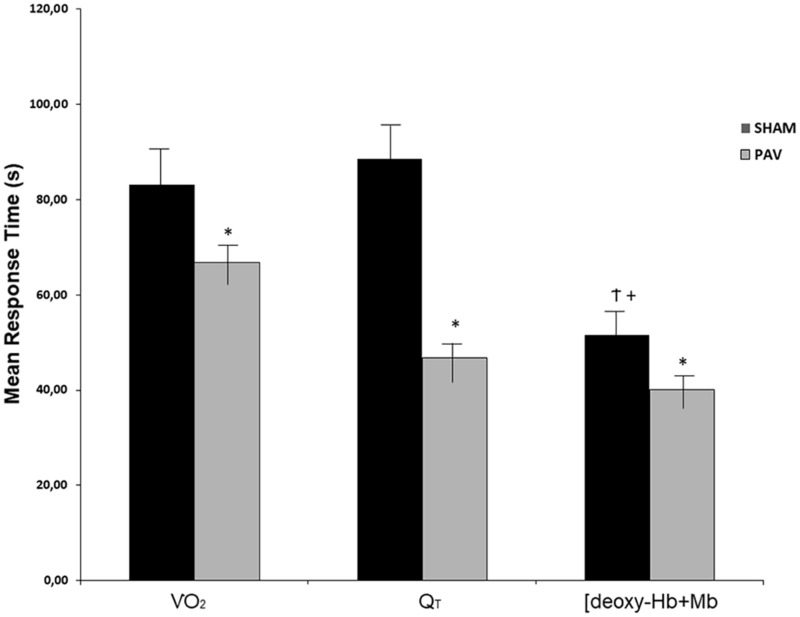
Mean response time (MRT) of ${\dot{\text{V}}\text{O}}_{2p}$, Q_*T*_, and deoxy-hemoglobin concentration ([deoxy-Hb + Mb]), on recovery of heavy-intensity exercise during Sham (open bars) and PAV (solid bars). Note that the dynamics of ${\dot{\text{V}}\text{O}}_{2p}$ and Q_*T*_ and [deoxy-Hb + Mb] recovery were faster during PAV (*p* < 0.05). In addition, [deoxy-Hb + Mb] kinetic was faster than QT and ${\dot{\text{V}}\text{O}}_{2p}$ only when Sham was administered in HFrEF patients. Values are means (SD). ^∗^*p* < 0.05 for between-intervention comparisons; ^†^*p* < 0.05 for within-variables comparisons between [deoxy-Hb + Mb] vs. ${\dot{\text{V}}\text{O}}_{2p}$; and ^+^*p* < 0.05 for within-group comparisons of [deoxy-Hb + Mb] vs. Q_*T*_.

**FIGURE 3:**
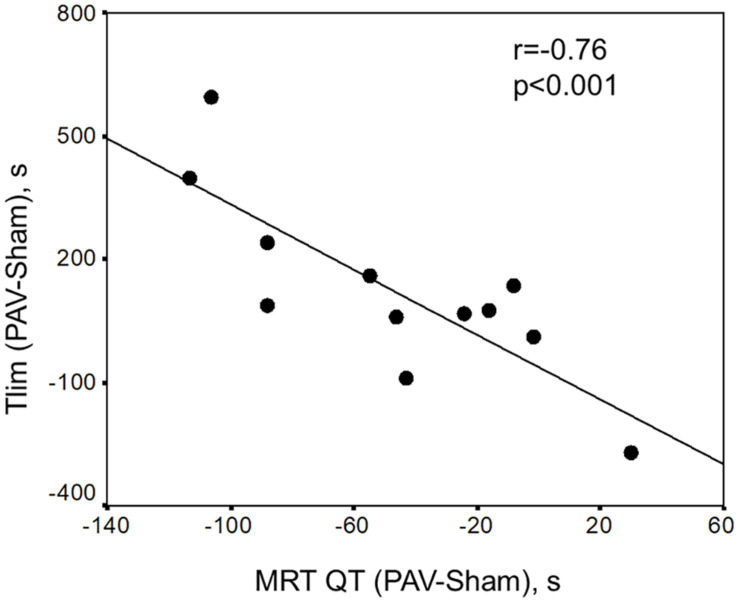
Significant inverse relationship between the difference of limit of tolerance with PAV-Sham vs. the difference of mean response time (MRT) of QT (PAV-Sham). These data suggest that the higher variation of Tlim with PAV, the faster lower “central” cardiovascular kinetics (Pearson correlation = 0.76, *p* < 0.001).

All fitted data were included in the kinetics analysis as *r*^2^-values ranged from 0.90 to 0.99. Off-exercise PAV accelerated $\dot{\text{V}}{\text{O}}_{2}$ kinetics when compared to sham ventilation (representative subject in Figure 1A and sample values in Table 3). In parallel, Q_*T*_ recovery kinetics was faster with PAV (Figure 1B and Table 3) (*p* < 0.05). The accelerated Q_*T*_ kinetics was largely explained by a faster HR recovery with PAV (Table 3). Similar speeding effects of PAV were observed in relation to [deoxy-Hb + Mb] (Figure 1C and Table 3). Consistent with these results, $\dot{\text{V}}{\text{O}}_{2}$, Q_*T*_, and [deoxy-Hb + Mb] MRT values were shorter with PAV compared to sham ventilation (Figure 2). The improvement in Q_*T*_ dynamics with active intervention was related to enhanced exercise tolerance (*p* < 0.001, Figure 3).

**TABLE 3:** Off-exercise kinetic parameters for oxygen uptake (${\dot{\text{V}}O}_{2p}$), [deoxy-Hb/Mb], and cardiac output (Q_*T*_) after sham or proportional assist ventilation (PAV) (*N* = 10).

Variables	Sham	PAV	*P-*level
**${\dot{\text{V}}\text{O}}_{2p}$**			
Baseline (ml)	1,224 ± 272	1,205 ± 289	0.12
A (ml)	952 ± 242	882 ± 235	0.76
*τ* (s)	77 ± 42	56 ± 22	0.04
***Q*_*T*_**			
Baseline (ml)	10 ± 2	10 ± 1	0.22
A (ml)	5 ± 1	5 ± 1	0.15
*τ* (s)	78 ± 46	39 ± 22	0.02
**HR**			
Baseline (ml)	109 ± 10	109 ± 15	0.95
A (ml)	31 ± 11	29 ± 7	0.48
*τ* (s)	54 ± 23	35 ± 13	0.01
MRT (s)	63 ± 22	41 ± 17	0.003
[deoxy-Hb + Mb]			
Baseline (ml)	53 ± 29	54 ± 30	0.86
A (ml)	78 ± 37	67 ± 26	0.55
*τ* (s)	42 ± 22	31 ± 19	0.04

**Values are means ± SD. Definition of symbols and abbreviations: TD, time delay; τ, time constant. $\dot{V}O_{2p}$, Pulmonary oxygen uptake, Q_*T*_, cardiac output, MRT, mean response time, [deoxy-Hb/Mb], deoxyhemoglobin + myoglobin concentration by NIRS.**

## Discussion

The novel findings of the present study in patients with stable, but advanced, HFrEF are as follows: (1) PAV improved exercise tolerance and ventilatory efficiency; (2) PAV accelerated the recovery of $\dot{\text{V}}{\text{O}}_{2}$, as well as [deoxy-Hb + Mb] (a non-invasive estimate of fractional O_2_ extraction) (Barstow, 2019), and central hemodynamics; and (3) a faster recovery of central hemodynamics with PAV was associated with better exercise tolerance. These data indicate that unloading the respiratory muscles has positive effects on O_2_ delivery to, and utilization by, the peripheral muscles during passive recovery from exercise in HFrEF. These results set the stage for future studies assessing a role for respiratory muscle unloading in enhancing the tolerance to repeated (interval) exercise in these patients.

### Effects of PAV on Muscle Reoxygenation Kinetics

It is widely recognized that the skeletal muscle deoxygenation at the onset of exercise in patients with HFrEF is related to impairments of local O_2_ delivery and utilization (Richardson et al., 2003). In addition, experimental evidence suggests that, as HFrEF progresses, there is a slower recovery of microvascular PO_2_ (PmvO_2_), reflected by impaired microvascular O_2_ delivery-to-utilization matching in the active muscle, i.e., lower PmvO_2_ (Copp et al., 2010). A lower PmvO_2_, in turn, may impair the recovery of intracellular metabolic homeostasis, delaying phosphocreatine resynthesis after exercise in HFrEF. These important metabolic changes increase muscle fatigability, likely impairing the ability to perform subsequent physical tasks (Krause et al., 2005; Copp et al., 2010). In this sense, ventilatory strategies that can reduce fatigability and increase muscle recovery for a new high-intensity task would be relevant for the cardiopulmonary rehabilitation of these patients. In addition, HFrEF may be associated with redistribution of an already-reduced cardiac output toward the respiratory muscles, leading to lower peripheral muscle perfusion and O_2_ supply. Collectively, these abnormalities may impair leg muscles’ oxidative capacity with negative effects on dyspnea, leg discomfort, and exercise tolerance in these patients (Poole et al., 2012).

In the present study, PAV accelerated the recovery of leg muscle oxygenation, as indicated by a faster decrease in [deoxy-Hb + Mb] (Table 3 and Figure 2). The explanation for this finding might be multifactorial. For instance, PAV may have increased peripheral vascular conductance *via* lower sympathetic outflow (Olson et al., 2010) in response to a lessened respiratory muscle metaboreflex (Sheel et al., 2018). In fact, this was previously shown that at a given Q_*T*_ and time, PAV was associated with increased oxygenation and higher blood flow to the appendicular musculature in patents with HFrEF, suggesting blood flow redistribution (Borghi-Silva et al., 2008b). Of note, Miller et al. found that decreasing the work of breathing with inspiratory positive pressure ventilation increased hindlimb blood flow out of proportion to increases in cardiac output in dogs with experimental HFrEF (Miller et al., 2007). Thus, bulk blood flow to the legs may have been enhanced by PAV despite a faster decrease in Q_*T*_, which would tend to *reduce* convective O_2_ delivery at a given time point. The positive effects of PAV on muscle blood flow during high-intensity exercise may have persisted throughout the recovery phase, leading to more pronounced post-exercise hyperemia (Goulart et al., 2020a).

A preferential distribution of local blood flow toward type II fibers, which are less efficient on O_2_ utilization compared to type I fibers, is also conceivable (Barstow et al., 1996; Poole et al., 2012). Another possible mechanism demonstrated is that under hypoxia conditions, [deoxy-Hb + Mb] occurs at a lower energy output (Rafael de Almeida et al., 2019). It should also be acknowledged that the positive effects of PAV on on-exercise $\dot{\text{V}}{\text{O}}_{2}$ kinetics (i.e., low O_2_ deficit) may have decreased O_2_ debt, leading to a faster decrease in off-exercise $\dot{\text{V}}{\text{O}}_{2}$ (Mazzuco et al., 2020). Consistent with the current findings, this study found that respiratory muscles unloading reduced leg fatigue during high-intensity isokinetic exercise, supporting evidence that this strategy might have an adjunct role to improve patients’ response to rehabilitative exercise in HFrEF (Borghi-Silva et al., 2009).

The QT off-kinetics were also accelerated with PAV (Table 3 and Figure 1). This might be related to the fact that PAV was associated with lower O_2_ demands during recovery, likely due to improved muscle bioenergetics, i.e., faster PCr resynthesis (Yoshida et al., 2013). Additionally, a lower sympathetic drive with non-invasive ventilation may have prompted a faster increase in parasympathetic tonus (Borghi-Silva et al., 2008c); in fact, the quicker decrease in Q_*T*_ was largely secondary to a faster HR recovery (Table 3). Interestingly, a strong correlation between faster Q_*T*_ decline and increases in Tlim with PAV was found (Figure 3). Again, this might reflect a larger decrease in sympathetic efference in patients who derived greater benefit from PAV. Additional studies quantifying sympathetic neural outflow at similar exercise duration with PAV and sham ventilation are warranted to confirm (or negate) this hypothesis (Borghi-Silva et al., 2008c; Reis et al., 2014).

### PAV and Ventilatory Efficiency in HFrEF

The present study found that respiratory muscle unloading with PAV was associated with improved ventilatory efficiency, i.e., lower ${\dot{\text{V}}}_{E}$–$\dot{\text{V}}{\text{CO}}_{2}$ relationship (Table 2). Of note, however, this was not a consequence of lower ${\dot{\text{V}}}_{E}$ at a given $\dot{\text{V}}{\text{CO}}_{2}$, but rather similar ${\dot{\text{V}}}_{E}$ *despite* a higher $\dot{\text{V}}{\text{CO}}_{2}$. Higher $\dot{\text{V}}{\text{CO}}_{2}$ (and, to a lesser extent, $\dot{\text{V}}{\text{O}}_{2}$) at exercise cessation with PAV than sham might reflect the effects of a longer test in the former intervention during the PAV trial. This may also occur due to the dynamics of $\dot{\text{V}}{\text{CO}}_{2}$ and its relationship with the kinetics of CO_2_ storage and production (Scott Bowen et al., 2012).

It remains unclear, however, why ${\dot{\text{V}}}_{E}$ remained unaltered despite a higher CO_2_ “load” since the respiratory neural drive, lung mechanics, or ventilation/perfusion (mis)matching was not assessed. Regardless of the mechanism, a reduction in the ${\dot{\text{V}}}_{E}$–$\dot{\text{V}}{\text{CO}}_{2}$ through pharmacological and non-pharmacological interventions may have relevant clinical implications, including improved survival (Paolillo et al., 2019). It is worth noting that dyspnea ratings at Tlim were similar between conditions despite a longer Tlim with PAV (Table 2). This might reflect the effects of an unaltered ${\dot{\text{V}}}_{E}$ and/or the beneficial consequences of inspiratory muscle unloading.

### Methodological Considerations and Potential Limitations

The present study focused on the effects of PAV on recovery kinetics since the presence of oscillatory ventilation in half of patients precluded the analysis of on-exercise $\dot{\text{V}}{\text{O}}_{2}$ kinetics (Sperandio et al., 2009). Consistent with these results, previous studies showed that recovery kinetics were more reproducible, being determined with a higher degree of reliability and validity (Kemps et al., 2009, 2010). Nevertheless, the present study acknowledges that by not repeating the exercise bout, it is limited in its ability to determine the actual beneficial effects of PAV on the tolerance of any ensuing exercise. It is reasoned that a second session could influence [deoxy-Hb + Mb] due to changes in probe position, thereby decreasing the between-days comparability. Moreover, this study did not measure the work of breathing; thus, the magnitude of respiratory muscle unloading brought by PAV in individual patients remains unclear. As a non-invasive study, it relied on signal-morphology cardioimpedance to measure Q_*T*_ (Borghi-Silva et al., 2008a; Paolillo et al., 2019). Although this method is not free from caveats (Wang and Gottlieb, 2006), it has provided acceptable estimates of changes in Q_*T*_ in patients with cardiopulmonary diseases (Vasilopoulou et al., 2012; Louvaris et al., 2019).

### Clinical Implications

The findings of the present study indicate that respiratory muscle unloading improves muscle oxygenation during recovery from high-intensity exercise, suggesting that non-invasive ventilation (PAV) might be used as an adjunct strategy to improve the tolerance to subsequent exercise during cardiac rehabilitation. Future studies could investigate the effects of such strategy in cardiopulmonary rehabilitation programs. It is conceivable that such an effect would be particularly relevant to more severe patients exposed to interval training (Spee et al., 2016) or, as described before, to strength training (Borghi-Silva et al., 2009). If the beneficial effects of PAV on muscle oxygenation prove to be associated with improved autonomic modulation (lower sympathetic drive), long-term respiratory muscle unloading may have an hitherto unexplored effect on other relevant outcomes in HFrEF, such as ventricular tachyarrhythmias, cardiac remodeling, and left ventricle afterload (Cornelis et al., 2016).

## Conclusion

Respiratory muscle unloading promoted by PAV improves leg muscle oxygenation during the recovery from high-intensity exercise in patients with HFrEF. These results add novel evidence that the salutary consequences of PAV on the physiological responses to dynamic exercise in HFrEF (Borghi-Silva et al., 2008a,b; Carrascossa et al., 2010) extend to the recovery phase, an effect that might be of practical relevance to improve tolerance to repeated (interval) exercise.

## Data Availability Statement

The raw data supporting the conclusions of this article will be made available by the authors, without undue reservation in the Institutional Repository of UFSCar.

## Ethics Statement

The studies involving human participants were reviewed and approved by the CEP 0844/06. Written informed consent to participate in this study was provided by the participants’ legal guardian/next of kin.

## Author Contributions

AB-S, CG, CC, CO, DB, DA, LN, RA, and JN: conceptualization, data curation, formal analysis, investigation, methodology, project administration, supervision, writing—original draft, and writing—review and editing. All authors contributed to the article and approved the submitted version.

## Conflict of Interest

The authors declare that the research was conducted in the absence of any commercial or financial relationships that could be construed as a potential conflict of interest.

## References

[B1] Bailey C. S. Wooster L. T. Buswell M. Patel S. Bakken K. White C. (2018). Post-Exercise Oxygen Uptake Recovery Delay: A Novel Index of Impaired Cardiac Reserve Capacity in Heart Failure. *JACC Hear. Fail.* 6 329–339. 10.1016/j.jchf.2018.01.007.Post-Exercise PMC5880321 29525330

[B2] Barstow T. J. (2019). Understanding near infrared spectroscopy and its application to skeletal muscle research. *J. Appl. Physiol.* 126 1360–1376. 10.1152/japplphysiol.00166.2018 30844336

[B3] Barstow T. J. Jones A. M. Nguyen P. H. Casaburi R. (1996). Influence of muscle fiber type and pedal frequency on oxygen uptake kinetics of heavy exercise. *J. Appl. Physiol.* 81 1642–1650. 10.1152/jappl.1996.81.4.1642 8904581

[B4] Bianchi L. Foglio K. Pagani M. Vitacca M. Rossi A. Ambrosino N. (1998). Effects of proportional assist ventilation on exercise tolerance in COPD patients with chronic hypercapnia. *Eur. Respir. J.* 11 422–427. 10.1183/09031936.98.11020422 9551748

[B5] Borghi-Silva A. Carrascosa C. Oliveira C. C. Barroco A. C. Berton D. C. Vilaça D. (2008a). Effects of respiratory muscle unloading on leg muscle oxygenation and blood volume during high-intensity exercise in chronic heart failure. *Am. J. Physiol. - Hear. Circ. Physiol.* 294 2465–2472. 10.1152/ajpheart.91520.2007 18375714

[B6] Borghi-Silva A. Di Thommazo L. Pantoni C. B. F. Mendes R. G. De Fátima Salvini T. Costa D. (2009). Non-invasive ventilation improves peripheral oxygen saturation and reduces fatigability of quadriceps in patients with COPD. *Respirology* 14 537–544. 10.1111/j.1440-1843.2009.01515.x 19386071

[B7] Borghi-Silva A. Oliveira C. C. Carrascosa C. Maia J. Berton D. C. Queiroga F. (2008b). Respiratory muscle unloading improves leg muscle oxygenation during exercise in patients with COPD. *Thorax* 63 910–915. 10.1136/thx.2007.090167 18492743

[B8] Borghi-Silva A. Reis M. S. Mendes R. G. Pantoni C. B. F. Simões R. P. Martins L. E. B. (2008c). Noninvasive ventilation acutely modifies heart rate variability in chronic obstructive pulmonary disease patients. *Respir. Med.* 102 1117–1123. 10.1016/j.rmed.2008.03.016 18585024

[B9] Carrascossa C. R. Oliveira C. C. Borghi-Silva A. Ferreira E. M. V. Maya J. Queiroga F. (2010). Haemodynamic effects of proportional assist ventilation during high-intensity exercise in patients with chronic obstructive pulmonary disease. *Respirology* 15 1185–1191. 10.1111/j.1440-1843.2010.01846.x 20920126

[B10] Compostella L. Russo N. Setzu T. Compostella C. Bellotto F. (2014). Exercise Performance of Chronic Heart Failure Patients in the Early Period of Support by an Axial-Flow Left Ventricular Assist Device as Destination Therapy. *Artif. Organs.* 38 366–373. 10.1111/aor.12172 24117945

[B11] Copp S. W. Hirai D. M. Ferreira L. F. Poole D. C. Musch T. I. (2010). Progressive chronic heart failure slows the recovery of microvascular O2 pressures after contractions in the rat spinotrapezius muscle. *Am. J. Physiol. - Hear. Circ. Physiol.* 299:2010. 10.1152/ajpheart.00590.2010 20817826 PMC3006296

[B12] Cornelis J. Beckers P. Taeymans J. Vrints C. Vissers D. (2016). Comparing exercise training modalities in heart failure: A systematic review and meta-analysis. *Int. J. Cardiol.* 221 867–876. 10.1016/j.ijcard.2016.07.105 27434363

[B13] da Luz Goulart C. Caruso F. R. Garcia, de Araújo A. S. Tinoco Arêas G. P. (2020). Non-invasive ventilation improves exercise tolerance and peripheral vascular function after high-intensity exercise in COPD-HF patients. *Respir. Med.* 173 106173. 10.1016/j.rmed.2020.106173 33007709

[B14] Dall’Ago P. Chiappa G. R. S. Guths H. Stein R. Ribeiro J. P. (2006). Inspiratory muscle training in patients with heart failure and inspiratory muscle weakness: A randomized trial. *J. Am. Coll. Cardiol.* 47 757–763. 10.1016/j.jacc.2005.09.052 16487841

[B15] Fortin M. Turgeon P. Y. Na0dreau É Grégoire P. Maltais L. G. Sénéchal M. (2015). Prognostic Value of Oxygen Kinetics During Recovery From Cardiopulmonary Exercise Testing in Patients With Chronic Heart Failure. *Can. J. Cardiol.* 31 1259–1265. 10.1016/j.cjca.2015.02.015 26115872

[B16] Francis D. P. Davies L. C. Willson K. Wensel R. Ponikowski P. Coats A. J. S. (2002). Impact of periodic breathing on measurement of oxygen uptake and respiratory exchange ratio during cardiopulmonary exercise testing. *Clin. Sci.* 103 543–552. 10.1042/cs1030543 12444906

[B17] Georgantas A. Dimopoulos S. Tasoulis A. Karatzanos E. Pantsios C. Agapitou V. (2014). Beneficial effects of combined exercise training on early recovery cardiopulmonary exercise testing indices in patients with chronic heart failure. *J. Cardiopulm. Rehabil. Prev.* 34 378–385. 10.1097/HCR.0000000000000068 24983706

[B18] Goulart C. Caruso F. Araújo A. Arêas G. Moura S. Catai A. (2020a). Non-invasive ventilation improves exercise tolerance and peripheral vascular function after high-intensity exercise in COPD-HF patients. *Respir. Med.* 173:1. 10.1016/j.rmed.2020.106173 33007709

[B19] Goulart C. da L. Arêas G. P. T. Caruso F. R. Araújo A. S. G. de Moura S. C. G. (2020b). Effect of high-intensity exercise on cerebral, respiratory and peripheral muscle oxygenation of HF and COPD-HF patients. *Hear. Lung.* 000 1–8. 10.1016/j.hrtlng.2020.06.013 32709499

[B20] Guazzi M. Tumminello G. Di Marco F. Fiorentini C. Guazzi M. D. (2004). The effects of phosphodiesterase-5 inhibition with sildenafil on pulmonary hemodynamics and diffusion capacity, exercise ventilatory efficiency, and oxygen uptake kinetics in chronic heart failure. *J. Am. Coll. Cardiol.* 44 2339–2348. 10.1016/j.jacc.2004.09.041 15607396

[B21] Hirai D. M. Craig J. C. Colburn T. D. Eshima H. Kano Y. Musch T. I. (2019). Skeletal muscle interstitial PO2 kinetics during recovery from contractions. *J. Appl. Physiol.* 127 930–939. 10.1152/japplphysiol.00297.2019 31369325 PMC6850987

[B22] Hunt S. A. Abraham W. T. Chin M. H. Feldman A. M. Francis G. S. Ganiats T. G. (2005). ACC/AHA 2005 Guideline Update for the Diagnosis and Management of Chronic Heart Failure in the Adult. *Circulation* 112 154–235. 10.1161/circulationaha.105.167586 16160202

[B23] Keller-Ross M. L. Bruce D. J. Rickey E. C. Michael J. J. John H. E. Timothy B. C. (2016). Improved Ventilatory Efficiency with Locomotor Muscle Afferent Inhibition is Strongly Associated with Leg Composition in Heart Failure. *Physiol. Behav.* 176 139–148. 10.1016/j.ijcard.2015.08.212.Improved PMC4656052 26397403

[B24] Kemps H. M. C. De Vries W. R. Hoogeveen A. R. Zonderland M. L. Thijssen E. J. M. Schep G. (2007). Reproducibility of onset and recovery oxygen uptake kinetics in moderately impaired patients with chronic heart failure. *Eur. J. Appl. Physiol.* 100 45–52. 10.1007/s00421-007-0398-7 17277937 PMC1914232

[B25] Kemps H. M. C. Schep G. Hoogsteen J. Thijssen E. J. M. De Vries W. R. Zonderland M. L. (2009). Oxygen uptake kinetics in chronic heart failure: Clinical and physiological aspects. *Netherlands Hear. J.* 17 238–244. 10.1007/BF03086254 19789686 PMC2711249

[B26] Kemps H. M. Schep G. Zonderland M. L. Thijssen E. J. De Vries W. R. Wessels B. (2010). Are oxygen uptake kinetics in chronic heart failure limited by oxygen delivery or oxygen utilization? *Int. J. Cardiol.* 142 138–144. 10.1016/j.ijcard.2008.12.088 19168233

[B27] Krause D. J. Hagen J. L. Kindig C. A. Hepple R. T. (2005). Nitric oxide synthase inhibition reduces the O2 cost of force development in rat hindlimb muscles pump perfused at matched convective O 2 delivery. *Exp. Physiol.* 90 889–900. 10.1113/expphysiol.2005.031567 16123049

[B28] Louvaris Z. Spetsioti S. Andrianopoulos V. Chynkiamis N. Habazettl H. Wagner H. (2019). Cardiac output measurement during exercise in COPD: A comparison of dye dilution and impedance cardiography. *Clin. Respir. J.* 13 222–231. 10.1111/crj.13002 30724023

[B29] Mazzuco A. Souza A. S. Goulart C. da L. Medeiros W. M. Sperandio P. A. (2020). Noninvasive Ventilation Accelerates Oxygen Uptake Recovery Kinetics in Patients With Combined Heart Failure and Chronic Obstructive Pulmonary Disease. *J. Cardiopulm. Rehabil. Prev.* 40 414–420. 10.1097/HCR.0000000000000499 33074848

[B30] Miller J. D. Smith C. A. Hemauer S. J. Dempsey J. A. (2007). The effects of inspiratory intrathoracic pressure production on the cardiovascular response to submaximal exercise in health and chronic heart failure. *Am. J. Physiol. - Hear. Circ. Physiol.* 292 580–592. 10.1152/ajpheart.00211.2006 16997896

[B31] Neder J. A. Nery L. E. Castelo A. Andreoni S. Lerario M. C. Sachs A. (1999). Prediction of metabolic and cardiopulmonary responses to maximum cycle ergometry: A randomised study. *Eur. Respir. J.* 14 1304–1313. 10.1183/09031936.99.14613049 10624759

[B32] Olson T. P. Joyner M. J. Dietz N. M. Eisenach J. H. Curry T. B. Johnson B. D. (2010). Effects of respiratory muscle work on blood flow distribution during exercise in heart failure. *J. Physiol.* 588 2487–2501. 10.1113/jphysiol.2009.186056 20457736 PMC2915522

[B33] Paolillo S. Veglia F. Salvioni E. Corrà U. Piepoli M. Lagioia R. (2019). Heart failure prognosis over time: how the prognostic role of oxygen consumption and ventilatory efficiency during exercise has changed in the last 20 years. *Eur. J. Heart Fail.* 21 208–217. 10.1002/ejhf.1364 30632680

[B34] Poole D. C. Hirai D. M. Copp S. W. Musch T. I. (2012). Muscle oxygen transport and utilization in heart failure: Implications for exercise (IN)tolerance. *Am. J. Physiol. - Hear. Circ. Physiol.* 302 1050–1063. 10.1152/ajpheart.00943.2011 22101528 PMC3311454

[B35] Rafael de Almeida A. Jorge E Béjar S. Erin Calaine I. Danilo I. Juan M. M. (2019). The effect of the fraction of inspired oxygen on the NIRS-derived deoxygenated hemoglobin “breakpoint” during ramp-incremental test. *Am. J. Physiol. Regul. Integr. Comp. Physiol.* 8:55. 10.1152/ajpregu.00291.2019 PMC7052603 31850819

[B36] Reis H. V. Borghi-Silva A. Catai A. M. Reis M. S. (2014). Impacto da CPAP sobre a tolerância ao exercício físico e a modulação simpatovagal de pacientes com insuficiência cardíaca crônica. *Brazilian J. Phys. Ther.* 18 218–227. 10.1590/bjpt-rbf.2014.0037 25003274 PMC4183494

[B37] Richardson T. E. Kindig C. A. Musch T. I. Poole D. C. (2003). Effects of chronic heart failure on skeletal muscle capillary hemodynamics at rest and during contractions. *J. Appl. Physiol.* 95 1055–1062. 10.1152/japplphysiol.00308.2003 12740313

[B38] Scott Bowen T. Cannon D. T. Begg G. Baliga V. Witte K. K. Rossiter H. B. (2012). A novel cardiopulmonary exercise test protocol and criterion to determine maximal oxygen uptake in chronic heart failure. *J. Appl. Physiol.* 113 451–458. 10.1152/japplphysiol.01416.2011 22653993 PMC3426168

[B39] Sheel A. W. Boushel R. Dempsey J. A. (2018). Competition for blood flow distribution between respiratory and locomotor muscles: Implications for muscle fatigue. *J. Appl. Physiol.* 125 820–831. 10.1152/japplphysiol.00189.2018 29878876 PMC6842878

[B40] Spee R. F. Niemeijer V. M. Wijn P. F. Doevendans P. A. Kemps H. M. (2016). Effects of high-intensity interval training on central haemodynamics and skeletal muscle oxygenation during exercise in patients with chronic heart failure. *Eur. J. Prev. Cardiol.* 23 1943–1952. 10.1177/2047487316661615 27440661

[B41] Sperandio P. A. Borghi-Silva A. Barroco A. Nery L. E. Almeida D. R. Neder J. A. (2009). Microvascular oxygen delivery-to-utilization mismatch at the onset of heavy-intensity exercise in optimally treated patients with CHF. *Am. J. Physiol. - Hear. Circ. Physiol.* 297 1720–1728. 10.1152/ajpheart.00596.2009 19734359

[B42] van der Zwaard S. Jaspers R. T. Blokland I. J. Achterberg C. Visser J. M. den Uil A. R. (2016). Oxygenation Threshold Derived from Near-Infrared Spectroscopy: Reliability and Its Relationship with the First Ventilatory Threshold. *PLoS One* 15:e0162914. 10.1371/journal.pone.0162914 27631607 PMC5025121

[B43] Vasilopoulou M. K. Vogiatzis I. Nasis I. Spetsioti S. Cherouveim E. Koskolou M. (2012). On- and off-exercise kinetics of cardiac output in response to cycling and walking in COPD patients with GOLD Stages I-IV. *Respir. Physiol. Neurobiol.* 181 351–358. 10.1016/j.resp.2012.03.014 22484002

[B44] Wang D. J. Gottlieb S. S. (2006). Impedance cardiography: More questions than answers. *Curr. Heart Fail. Rep.* 3 107–113. 10.1007/s11897-006-0009-7 16914102

[B45] Yoshida T. Abe D. Fukuoka Y. (2013). Phosphocreatine resynthesis during recovery in different muscles of the exercising leg by 31P-MRS. *Scand. J. Med. Sci. Sport.* 23 1–7. 10.1111/sms.12081 23662804

[B46] Younes M. (1992). Proportional assist ventilation, a new approach to ventilatory support: Theory. *Am. Rev. Respir. Dis.* 145 114–120. 10.1164/ajrccm/145.1.114 1731573

